# Electrochemical behavior and in-vitro antimicrobial screening of some thienylazoaryls dyes

**DOI:** 10.1186/s13065-017-0345-6

**Published:** 2017-11-21

**Authors:** Joseph Tsemeugne, Emmanuel Sopbué Fondjo, Jean-de-Dieu Tamokou, Ignas Tonle, Irene Chinda Kengne, Arnaud Djintchui Ngongang, Stephen Tamekou Lacmata, Taoufik Rohand, Jules Roger Kuiate, Beibam Luc Sondengam

**Affiliations:** 1 Laboratory of Applied Synthetic Organic Chemistry, Department of Chemistry, Faculty of Science, University of Dschang, P.O. Box 67, Dschang, Republic of Cameroon; 2 Department of Organic Chemistry, University of Yaounde I, P.O. Box 812, Yaounde, Republic of Cameroon; 3 Laboratory of Microbiology and Antimicrobial Substances, Department of Biochemistry, Faculty of Science, University of Dschang, PO Box 067, Dschang, Republic of Cameroon; 4 Laboratory of Analytical and Molecular Chemistry, Faculty Polydisciplinaire of Safi, University Cadi Ayyad Marrakech, Route Sidi Bouzid BP 4162, Safi, 46000 Morocco

**Keywords:** Azo compounds, Carbon paste electrode, Cyclic voltammetry, Antibacterial agents, Antifungal agents

## Abstract

**Background:**

A series of recently reported phenolic azo dyes **7a–e** were prepared by coupling the thienyl diazonium sulfate of 3-Amino-4H-benzo[f]thieno[3,4-c](2H)chromen-4-one with selected diversely substituted phenolic and naphtholic derivatives. These compounds were evaluated for their antibacterial and antifungal activities. Furthermore their voltammetric behavior was compared at a glassy carbon electrode.

**Results:**

The voltammetric behavior of the five recently reported azo dyes has been compared at a glassy carbon electrode. It is shown that the azo dyes **7a–e** with a hydroxyl group in the *ortho* position with respect to the azo bridge give rise to well defined, irreversible peaks for the oxidation and reduction process within a pH range of 2–7. The mechanisms of electrochemical oxidation of compound **7a**–**c** and **7e** are proposed. For the hydroxyl-substituted dyes, re-oxidation peaks were obtained in the subsequent scan. The antimicrobial activities of the reported compounds **7a–e** along with the entire precursors **1–4** and **6a–e** were performed against selected bacterial and fungal species and their activities compared to those of nystatin, griseofulvin and ciprofloxacin used as reference drugs.

**Conclusions:**

The present study showed significant antimicrobial activity of compounds **6d**, **7a** and **7c,e** against the tested microorganisms; this result confirms the antimicrobial potency of azo compounds and some of their precursors.

## Introduction

Before the discovery of the first synthetic organic dye in 1856, namely the Mauvaine [1], human being has to use some part of plants like roots and leaves to color textile fibers. This discovery will kick off a new area in the field of dye research and nowadays approximately all molecules used as dyes in the textile industry are synthetic with a strong fondness for diazo dyes [2–4]. In addition to this traditional application of dyes, other fields of interest make azo compounds a real source of hope, notably in pharmaceutical [5–7] and food industries [8, 9], and in dosimetry [10, 11]. In general, azo dyes are highly preferred because they attach to the fibers by forming strong covalent bonds with the hydroxyl groups of the cellulose [12], so that the process of discoloration is very difficult under the conditions where textiles are washed [13, 14]. It is also demonstrated that the degradation of the textile is linked to the action of the microorganisms that are attached to it and it is to remedy this problem that the textile industry makes increasing use of biocidal dyes such as diazo dyes [15]. The textile industry is also increasingly faced with the problem of treating wastewater polluted by the rest of the dyes that have not been fixed to the fibers during the coloring process [16]. Due to their structural complexity, azo dyes often have very low biodegradability and their biological treatments are usually expensive and generally inefficient [17]. For this purpose, electrochemical techniques are the most recommended alternative because of their high efficiency, their eco-friendliness and their relatively low cost [17]. We recently reported the synthesis of a cyclic holigomeric azo dye containing three –N=N– units alternating with three fused thienocoumarins moieties in its structure and found to possess antioxidant properties. This means that it is capable to reduce an oxidant such as Fe^3+^ into Fe^2+^ through its diazo functionalities [18], justifying thereby the use of diazo compounds in chemotherapy treatments [19, 20]. In addition, encouraged by previous promising results obtained from the biological activities studies of some of the azo compounds that we have recently reported [21], we therefore undertook to intensify our research for new azo compounds with good dyeing [5, 22], biological properties [6, 23], and which could also find interesting applications in solving some issues related to industrial environmental pollution [16]. In this work, we investigated the antimicrobial activities of five recently reported [24] azo compounds respectively on six bacterial and six fungal strains. On the other hand, in order to assess their possible applications in the textile industry waste water remediation, and the evidence concerning the mechanisms of biological electron-transfer processes we carried out the electrochemical characterization of these compounds, on the carbon electrode.

## Experimental

### General information

All melting points were corrected and were determined using an Electrothermal Melting Point Apparatus Model 9100, a Büchi 530 melting point apparatus and a Stuart Scientific Melting Point Apparatus SMP3. The Thin Layer Chromatography (TLCs) was carried out on Eastman Chromatogram Silica Gel Sheets (13,181; 6060) with fluorescent indicators. A mixture of ethyl acetate and methylene chloride (7:3) was used as the eluent and iodine was used for the visualization of the chromatograms. The IR spectra were measured with a Fourier Transform Infrared spectrometer JASCO FT/IR-4100 and a Perkin Elmer FT-IR 2000 spectrometer. The UV spectra were recorded with a Beckman U-640 Spectrophotometer, using samples’ solutions of concentration 2 × 10^−5^ mol L^−1^. Combustion analyses were carried out with a Euro EA CHNSO analyser from Hekatech company, and the results were found to be in good agreement (± 0.3%) with the calculated values. HREIMS were measured on mass spectrometer LCQ Classic with ESI Source from Thermo Fisher Scientific Company. ^1^H-NMR spectra were recorded in DMSO-*d*_*6*_ with a 250 MHz spectrometer Bruker AV III. ^13^C-NMR spectra were recorded in DMSO-*d*_*6*_ with a 62.5 MHz spectrometer Bruker AV III. Tetramethyl silane (TMS) was used as the internal reference.

### Preparation of the reagents and starting materials

All the reagents mentioned in this work were purchased from Aldrich and Fluka and were used without further purification. Starting material **4** was prepared according to the procedures mentioned in the literature published earlier [25].

### Preparation of diazonium salt solution

In a similar manner as described in [24] dry sodium nitrite (2.07 g, 3 mmol) was slowly added over a period of 30 min to concentrated sulphuric acid (10 mL) with occasional stirring. The solution was cooled to 0–5 °C. Compound **4** was dissolved in DMSO (10 mL) and cooled to 0–5 °C. The nitrosyl sulphuric acid solution kept at 0–5 °C was added to the solution of **4** and the temperature was maintained between 0 and 5 °C. The clear diazonium salt solution thus obtained consisting of the in situ-formed intermediate **5**, was used immediately in the coupling reactions.

### General procedure for the preparation of the coupling products 7a–e

Phenol derivatives **6a–e** (3 mmol) were dissolved in DMSO (10 mL) and then cooled in an ice-bath at 0–5 °C. The diazonium solution of **4** previously prepared was added drop wise over 1 h, and then 15 mL of sodium acetate solution (10%) was added to the mixture. The pH of the mixtures was in the range 9–11. The solid precipitate was collected on a filter and crystallised from methanol to give the title compounds **7**. The freshly prepared compounds were characterized by their physical, elemental and spectroscopic data which were found to be in full agreement with those published earlier [24].

#### *3*-*[2*-*(2*-*hydroxy*-*1*-*naphthyl)diazenyl]*-*4H*-*benzo[f]thieno[3,4*-*c]chromen*-*4*-*one dihydrate*, **7a**

Reaction of diazonium salt of **4** with **6a** gave compound **7a** as a red powder; yield 59%, 0.41 g, mp 185.7 °C; IR (KBr) ν_max_: 3558, 3544 (OH), 3164, 3153 (Ar. C–H), 1718 (C=O), 1617, 1439, 1412 (N = N) cm^−1^. UV (THF) λ_max_/nm (log ε): 242 (4.54), 254 (4.61), 277 (2.46), 290 (4.19), 298 (4.11), 335 (4.49), 356 (4.54), 374 (4.64), 391 (4.56). ^1^H NMR (250 MHz, DMSO-*d*_*6*_): δ_H_ 7.21 (1H, s, 1-H), 7.61 (1H, d, *J* 8.9 Hz, 6-H), 8.37 (1H, d, *J* 8.8 Hz, 7-H), 8.13 (1H, d, *J* 8.0 Hz, 8-H), 7.71 (1H, dd, *J* 7.5 and 7.5 Hz, 9-H), 7.76 (1H, dd, *J* 7.5 and 7.8 Hz, 10-H), 8.63 (1H, d, *J* 9.0 Hz, 11-H), 7.75 (1H, d, *J* 8.0 Hz, 3′-H), 7.98 (2H, d, *J* 8.0 Hz, 4′-H and 5′-H), 7.50 (1H, dd, *J* 7.5 and 7.0 Hz, 6′-H), 8.00 (1H, dd, *J* 8.8 and 8.1 Hz, 7′-H), 7.33 (1H, d, *J* 8.8 Hz, 8′-H), 2.51 (1H, D_2_O-exchangeable, OH). ^13^C NMR (62.50 MHz, DMSO-*d*_*6*_): *δ*_*C*_ 118.2 (C-1), 134.3 (C-3), 116.3 (C-3a), 166.4 (C-4), 156.8 (C-5a), 125.6 (C-6 and C-3′), 139.5 (C-7), 131.4 (C-7a), 130.2 (C-8 and C-11b), 117.3 (C-9), 129.9 (C-10), 126.8 (C-11), 137.8 (C-1′ and 11a), 103.3 (C-11c), 158.5 (C-2′), 127.8 (C-4′), 129.6 (C-4a′), 128.7 (C-5′), 118.9 (C-6′), 132.7 (C-7′), 120.8 (C-8′), 147.7 (C-8a′). MS, *m/z* (%) = 236 (47), 288 (30), 296 (100), 373 (40), 393 (50), 328 (100), 340 (22), 421 (22). Anal. Calcd for C_25_H_18_N_2_O_5_S (458.49): C, 65.50; H, 3.93; N, 6.11; S, 6.98. Found: C, 65.48; H, 3.91; N, 6.09; S, 6.96.

#### *3*-*[2*-*(4*-*acetyl*-*3*-*hydroxy*-*2*-*naphthyl)diazenyl]*-*1*-*[2*-*(4*-*oxo*-*4H*-*benzo[f]thieno[3,4*-*c]chromen*-*3*-*yl)diazenyl]*-*4H*-*benzo[f]thieno[3,4*-*c]chromen*-*4*-*one disulphate dihydrate*, **7b**

Reaction of diazonium salt of **4** with **6b** gave compound **7b** as an orange powder; yield 28%, 0.38 g, mp 194.9 °C; IR (KBr) ν_max_: 3280(OH), 3076 (Ar. C–H), 1720 (C=O), 1619, 1529, 1438 (N=N) cm^−1^. UV (THF) λ_max_/nm (log ε): 240 (4.34), 253 (4.43), 293 (3.95), 328 (4.25), 343 (4.20), 360 (4.31), 373 (4.39), 388 (4.28). ^1^H NMR (250 MHz, DMSO-*d*_*6*_): δ_H_ 3.45 (3H, s, CH_3_), 6.94 (1H, s, H-1′′), 7.41 (OH, broad s, D_2_O-exchangeable), 7.12 (1H, s, H-1′), 8.52 (1H, dd, *J* 10.53 and 10.50 Hz, H-9), 8.35 (1H, dd, *J* 7.75 and 6.50 Hz, H-9′′), 8.00 (1H, dd, *J* 9.54 and 7.50 Hz, H-10), 7.88 (1H, dd, *J* 8.75 and 7.45 Hz, H-6′), 7.75 (1H, dd, *J* 10.50 and 10.45 Hz, H-7′), 7.62 (1H, dd, *J* 8.00 and 7.70 Hz, H-10′′), 9.01 (1H, d, *J* 7.50 Hz, H-11), 8.91 (1H, d, *J* 8.70 Hz, H-5′), 8.75 (1H, d, *J* 8.00 Hz, H-11′′), 8.18 (1H, d, *J* 8.00 Hz, H-6′′), 7.67 (1H, d, *J* 11.50 Hz, H-8′), 7.55 (1H, d, *J* 8.00 Hz, H-7′′), 7.51 (1H, d, *J* 10.00 Hz, H-8), 7.50 (1H, d, *J* 9.00 Hz, H-7), 7.44 (1H, d, *J* 8.00 Hz, H-8′′), 7.33 (1H, d, *J* 9.00 Hz, H-6). ^13^C NMR (62.50 MHz, DMSO-*d*_*6*_): *δ*_*C*_ 164.2 (CO), 134.7 (C-1), 145.0 (C-3), 115.4 (C-3a), 157.0 (C-4), 148.5 (5a), 120.0 (C-6), 130.7 (C-7), 133.7 (C-7a), 131.6 (C-8), 122.2 (C-9), 127.8 (C-10), 124.8 (C-11), 131.0 (C-11a), 103.8 (C-11b), 114.3 (C-11c), 121.0 (C-1′), 137.0 (C-2′), 155.0 (C-3′), 113.7 (C-4′), 122.7 (C-4a′), 133.0 (C-5′), 127.8 (C-6′), 126.2 (C-7′), 128.5 (C-8′), 103.2 (C-8a′), 119.0 (C-1′′), 139.1 (C-3′′), 115.0 (C-3a′′), 156.6 (C-4′′), 148.1 (5a′′), 123.6 (C-6′′), 129.6 (C-7′′), 133.2 (C-7a′′), 119.8 (C-8′′), 121.0 (C-9′′), 128.8 (C-10′′), 126.0 (C-11′′), 130.8 (C-11a′′), 103.5 (C-11b′′), 114.0 (C-11c′′), 33.0 (CH_3_). MS, *m/z* (%) = 237 (42), 250 (62), 258 (100), 276 (45), 313 (17), 373 (80), 404 (25), 521 (22), 644 (40), 660 (10). Anal. Calcd for C_42_H_30_N_4_O_16_S_4_ (974.96): C, 51.74; H, 3.10; N, 5.75; S, 13.16. Found: C, 51.65; H, 3.08; N, 5.80; S, 13.18.

#### *3*-*(2*-*{3*-*acetyl*-*2*-*hydroxy*-*5,6*-*bis[2*-*(4*-*oxo*-*4H*-*benzo[f]thieno[3,4*-*c]chromen*-*3 yl)diazenyl]phenyl}diazenyl)*-*4H*-*benzo[f]thieno[3,4*-*c]chromen*-*4*-*one trisulphate*, **7c**

Reaction of diazonium salt of **4** with **6c** gave compound **7c** as an orange powder; yield 26%, 0.65 g, mp 200.2 °C; IR (KBr) ν_max_: 3547 (OH), 1730 (C=O), 1517, 1435 (N=N) cm^−1^. UV (THF) λ_max_/nm (log ε): 236 (5.33), 267 (5.01), 286 (4.98), 335 (5.18), 438 (4.28), 540 (3.33). ^1^H NMR (250 MHz, DMSO-*d*_*6*_): δ_H_ 9.12 (1H, br s, H-4′), 9.00 (1H, d, *J* 8.75 Hz, H-7″), 8.83 (1H, d, *J* 9.50 Hz, H-7′″), 8.60 (1H, dd, *J* 8.75 Hz and *J’* 6.75 Hz, H-9″), 8.54 (1H, dd, *J* 9.50 Hz and *J* 6.75 Hz, H-10′″), 8.52 (1H, d, *J* 11.75 Hz, 8′″), 8.50 (1H, s, H-1″), 8.35 (1H, d, *J* 8.75 Hz, H-6″), 8.12 (1H, d, *J* 8.75 Hz, H-8″), 7.95 (1H, d, *J* 8.25 Hz, H-11″), 7.93 (1H, d, *J* 12.75 Hz, H-7), 7.88 (1H, dd, *J* 8.00 Hz and *J’* 7.75 Hz, H-9), 7.82 (1H, dd, *J* 8.50 Hz and *J’* 8.25 Hz, H-10″), 7.81 (1H, s, H-1), 7.78 (1H, d, *J* 12.75 Hz, H-6), 7.73 (1H, dd, *J* 7.50 Hz and *J’* 6.50 Hz, H-9′″), 7.74 (1H, d, *J* 12.00 Hz, H-11′″), 7.66 (1H, dd, *J* 13.00 Hz and *J*′ 7.25 Hz, H-10), 7.60 (1H, s, H-1′″), 7.58 (1H, d, *J* 9.00 Hz, H-6′″), 7.91 (1H, d, *J* 12.75 Hz, H-11), 7.95 (1H, d, *J* 8.25 Hz, H-8), 2.42 (3H, s, CH_3_-CO). ^13^C NMR (62.50 MHz, DMSO-*d*_*6*_): *δ*_*C*_ 125.8 (C-1), 132.6 (C-3), 114.4 (C-3a), 164.4 (C-4), 156.5 (C-5a), 121.8 (C-6), 137.8 (C-7), 131.5 (C-7a), 128.0 (C-8), 118.0 (C-9), 120.8 (C-10), 131.1 (C-11), 115.4 (C-11a), 148.2 (C-11b), 102.0 (C-11c), 133.0 (C-1′), 157.0 (C-2′), 122.6 (C-3′), 131.0 (C-4′), 138.2 (C-5′), 157.3 (C-6′), 126.3 (C-1″), 155.0 (C-3″), 113.5 (C-3a″), 165.0 (C-4″), 156.5 (C-5a″), 119.5 (C-6″), 130.3 (C-7″), 131.0 (C-7a″), 127.5 (C-8″), 117.8 (C-9″), 123.6 (C-10″), 129.5 (C-11″), 114.4 (C-11a″), 147.6 (C-11b″), 100.4 (C-11c″), 126.8 (C-1′″), 156.1 (C-3′″), 113.8 (C-3a′″), 163.7 (C-4′″), 155.3 (C-5a′″), 120.0 (C-6′″), 136.8 (C-7′″), 131.5 (C-7a′″), 131.5 (C-8′″), 132.1 (C-9′″), 138.7 (C-10′″), 136.2 (C-11′″), 103.7 (C-11a′″), 115.0 (C-11b′″), 100.5 (C-11c′″), 165.0 (COCH_3_), 25.8 (COCH_3_). MS, *m/z* (%) = 234 (23), 242 (63), 341 (74), 361 (54), 405 (17), 460 (100), 480 (24), 525 (10), 582 (9), 602 (4). Anal. Calcd for C_53_H_32_N_6_O_20_S_6_ (1265.24): C, 50.31; H, 2.55; N, 6.64; S, 15.21. Found: C, 50.29; H, 2.54; N, 6.62; S, 15.23.

#### *3*-*(2*-*{3*-*(tert*-*butyl)*-*2*-*hydroxy*-*5*-*methoxy*-*4,6*-*bis[2*-*(4*-*oxo*-*4H*-*benzo[f]thieno[3,4*-*c]chromen*-*3*-*yl)diazenyl]phenyl}diazenyl)*-*4H*-*benzo[f]thieno[3,4*-*c]chromen*-*4*-*one sulphate monohydrate*, **7d**

Reaction of diazonium salt of **4** with **6d** gave compound **7d** as a red powder; yield 19%, 0.43 g, mp 214.8 °C; IR (KBr) ν_max_: 3310 (OH), 3056 (Ar. C–H), 2960 (C_sp3_-H), 1734 (C=O), 1617, 1480, 1458 (N=N) cm^−1^. UV (THF) λ_max_/nm (log ε): 249 (5.34), 252 (5.34), 290 (4.94), 335 (4.52), 356 (4.44), 372 (4.46). ^1^H NMR (250 MHz, DMSO-*d*_*6*_): δ_H_ 7.65 (1H, s, 1-H″), 7.81 (1H, m, 6-H″), 8.64 (1H, m, 7-H″), 8.97 (1H, d, *J* 8.0 Hz, 8-H″), 7.87 (1H, d, *J* 7.5 Hz, 9-H″), 8.46 (1H, dd, *J* 7.5 and 8.3 Hz, 10-H″), 7.77 (1H, m, 11-H″), 7.84 (1H, s, 1-H), 7.93 (1H, d, *J* 8.0 Hz, 6-H), 8.58 (1H, d, *J* 9.3 Hz, 7-H), 8.76 (1H, dd, *J* 7.0 and 8.6 Hz, 8-H), 7.80 (1H, dd, *J* 7.5 and 7.5 Hz, 9-H), 8.34 (1H, m, 10-H), 7.90 (1H, d, *J* 8.0 Hz, 11-H), 7.90 (1H, s, 1′′′-H), 7.73 (1H, d, *J* 8.0 Hz, 6′′′-H), 8.32 (1H, m, 7′′′-H), 8.10 (1H, m, 8′′′-H), 8.00 (1H, d, *J* 6.8 Hz, 9′″-H), 8.44 (1H, m, 10′″-H), 8.38 (1H, m, 11′″-H), 3.60 (3H, s, OCH_3_), 3.21 (9H, s, CH_3_). ^13^C NMR (62.50 MHz, DMSO-*d*_*6*_): *δ*_*C*_ 126.6 (C-1), 147.5 (C-3), 113.0 (C-3a), 163.2 (C-4), 156.7 (C-5a), 121.0 (C-6), 137.6 (C-7), 130.7 (C-7a), 128.8 (C-8), 118.3 (C-9), 122.6 (C-10), 130.2 (C-11), 154.8 (C-11a), 115.3 (C-11b), 102.1 (C-11c), 114.6 (C-1′), 149.0 (C-2′), 121.5 (C-3′), 119.1 (C-4′), 138.3 (C-5′), 102.0 (C-6′), 126.8 (C-1″), 147.5 (C-3″), 113.2 (C-3a″), 164.3 (C-4″), 156.1 (C-5a″), 119.4 (C-6″), 135.9 (C-7″), 131.2 (C-7a″), 127.2 (C-8″), 117.6 (C-9″), 123.6 (C-10″), 129.4 (C-11″), 155.0 (C-11a″), 114.6 (C-11b″), 101.0 (C-11c″), 126.1 (C-1′″), 148.0 (C-3′″), 114.4 (C-3a′″), 163.2 (C-4′″), 155.8 (C-5a′″), 121.0 (C-6′″), 137.2 (C-7′″), 131.5 (C-7a′″), 128.0 (C-8′″), 117.6 (C-9′″), 122.0 (C-10′″), 130.0 (C-11′″), 138.3 (C-11a′″), 115.2 (C-11b′″), 103.7 (C-11c′″), 56.0 (OCH_3_), 18.6 (3CH_3_), 25.7 (C(CH_3_)_3_). MS, *m/z* (%) = 1131 (7), 1113 (21), 1015 (4), 985 (5), 852 (17), 799 (100), 754 (54), 736 (30), 275 (39), 261 (100). Anal. Calcd for C_56_H_38_N_6_O_13_S_4_ (1131.19): C, 59.46; H, 3.39; N, 7.43; S, 11.34. Found: C, 59.45; H, 3.41; N, 7.45; S, 11.35.

#### *3*-*{2*-*[3*-*(tert*-*butyl)*-*4*-*hydroxy*-*5*-*methylphenyl]diazenyl}*-*4H*-*benzo[f]thieno[3,4*-*c]chromen*-*4*-*one dihydrate*, **7e**

Reaction of diazonium salt of **4** with **6e** gave compound **7e** as a red powder; yield 69%, 0.33 g, mp 217.6 °C; IR (KBr) ν_max_: 3177 (Ar. C–H), 2954 (C_sp3_-H), 1718 (C=O), 1633, 1479, 1454 (N=N) cm^−1^. UV (THF) λ_max_/nm (log ε): 241 (4.50), 259 (4.51), 290 (4.18), 398 (5.05), 403 (5.05), 409 (5.06), 441.5 (5.06), 423 (5.03). ^1^H NMR (250 MHz, DMSO-*d*_*6*_): δ_H_ 7.23 (1H, s, 1-H), 7.48 (1H, d, *J* 7.8 Hz, 6-H), 8.34 (1H, d, *J* 7.8 Hz, 7-H), 8.50 (1H, d, *J* 7.7 Hz, 8-H), 8.33 (1H, dd, *J* 7.5 and 7.0 Hz, 9-H), 7.30 (1H, dd, *J* 6.7 and 7.4 Hz, 10-H), 8.68 (1H, d, *J* 7.5 Hz, 11-H), 7.65 (1H, s, 2′-H), 7.30 (1H, s, 6′-H), 3.56 (3H, s, CH_3_), 3.01 (9H, s, CH_3_). ^13^C NMR (62.50 MHz, DMSO-*d*_*6*_): *δ*_*C*_ 119.2 (C-1), 132.4 (C-3), 115.6 (C-3a), 164.5 (C-4), 155.2 (C-5a), 126.3 (C-6), 139.7 (C-7), 133.1 (C-7a), 130.1 (C-8), 120.7 (C-9), 128.0 (C-10), 125.5 (C-11), 135.2 (C-11a), 130.0 (C-11b), 104.0 (C-11c), 114.5 (C-1′), 119.3 (C-2′), 122.4 (C-3′), 148.7 (C-4′), 105.8 (C-5′), 108.8 (C-6′), 12.8 (CH_3_), 18.8 (3CH_3_), 26.0 (C(CH_3_)_3_). MS, *m/z* (%) = 1347 (70), 1231 (2), 827 (4), 726 (100), 663 (17), 325 (100). Anal. Calcd for C_26_H_26_N_2_O_5_S (478.56): C, 65.25; H, 5.48; N, 5.85; S, 6.70. Found: C, 65.23; H, 5.51; N, 5.84; S, 6.72.

### Cyclic voltammetry

Voltammetric measurements were carried out using a µ-Autolab (Ecochemie, Holland) controlled by the GPES electrochemical software. The working electrode was a glassy carbon electrode (0.3 mm diameter) properly polished using alumina paste prior to experiments. A Platinum gauze and a Ag/AgCl (3 M KCl) were used as counter and reference electrodes, respectively. Cyclic voltammograms were obtained with a scan rate of 50 mV s^−1^. Experiments were carried out at room temperature and in the presence of 0.1 M KCl or 0.02 M H_2_SO_4_ as supporting electrolyte, unless otherwise stated.

### Biological assay

#### Bacterial strains and culture media

The studied microorganisms were both reference (from the American Type Culture Collection) and clinical (from *Institut Pasteur* and *Ecole Nationale Vétérinaire d’Alford, France*) strains of *Providencia stuartii, Escherichia coli, Pseudomonas aeruginosa, Enterobacter aerogenes, Klebsiella pneumoniae, Candida albicans, Crytococcus neoformans* and *Trichophyton terrestre*. Also, included were two clinical isolates of *Trichophyton ajeloi* and *Trichophyton violaceum,* obtained at the Laboratory of Microbiology and Antimicrobial Substances, University of Dschang and two clinical isolates of *Candida parapsilosis* and *Staphylococcus aureus collected* from Pasteur Centre (Yaounde-Cameroon). The bacterial and fungal species were grown at 37/28 °C and maintained on nutrient agar (NA, Conda, Madrid, Spain) and Sabouraud Dextrose Agar (SDA, Conda) slants respectively.

#### Preparation of microbial inoculum

The inocula of yeasts and bacteria were prepared from overnight cultures by picking numerous colonies and suspending them in sterile saline (NaCl) solution (0.90%). Absorbance was red at 530 nm for yeasts or at 600 nm for bacteria. Adjustment was done with a saline solution to match that of a 0.50 McFarland standard solution. From the prepared microbial solutions, other dilutions with saline solution were prepared to give a final concentration of 10^6^ yeast cells/ml and 10^6^ CFU/ml for bacteria [19, 26].

Conidia suspensions of dermatophyte species were prepared from 10 days old cultures respectively. The number of conidia was determined using a spectrophotometer and adjusted with sterile saline (NaCl) solution (0.90%) to an absorbance of 0.600 at 450 nm corresponding to a final concentration of about 1 × 10^5^ spores/ml [27].

### Antimicrobial activity

The antimicrobial activity was investigated by determining the minimum inhibitory concentrations (MICs), minimum bactericidal concentrations (MBC) and minimum fungicidal concentrations (MFCs).

MICs were determined by broth micro dilution [28, 29]. Stock solutions of the pure compounds were prepared in 10% v/v aqueous dimethylsulfoxide (DMSO) solution (Fisher chemicals, Strasbourg, France) at concentration of 1024 µg/ml. This was twofold serially diluted in Mueller-Hinton Broth (MHB) for bacteria and Sabouraud Dextrose Broth (SDB) for fungi to obtain a concentration range of 512–0.25 µg/ml. For every experiment, a sterility check (10% aqueous DMSO and medium), negative control (10% aqueous DMSO, medium and inoculum) and positive control (10% aqueous DMSO, medium, inoculum and water-soluble antibiotics) were included. One hundred microliters of each concentration was introduced into a well (96-wells microplate) containing 90 µl of SDB or MHB and 10 µl of inoculum was added to obtain a final concentration range of 256–0.125 µg/ml. The plates were covered with a sterile lid, and incubated on the shaker at 37 °C for 24 h (bacteria), 48 h (yeasts) or 5 days (dermatophytes). MICs were assessed visually after the corresponding incubation period and were taken as the lowest sample concentration at which there was no growth or virtually no growth. The assay was repeated thrice.

For the minimum microbicidal concentration (MMC) determination, 10 µl aliquots from each well that showed no growth of microorganism were plated on Mueller-Hinton Agar or Sabouraud Dextrose Agar and incubated at 37 °C for 24 h (bacteria), 48 h (yeasts) and at 28 °C for 10 days (dermatophytes). The lowest concentration that yielded no growth after the sub-culturing was taken as the MBCs or MFCs. Ciprofloxacin (Sigma-Aldrich, Steinheim, Germany) for bacteria, nystatin (Sigma-Aldrich, Steinheim, Germany) for yeasts and griseofulvin (Sigma-Aldrich, Steinheim, Germany) for dermatophytes were used as positive controls.

## Results and discussion

### Chemistry

**Scheme 1:**
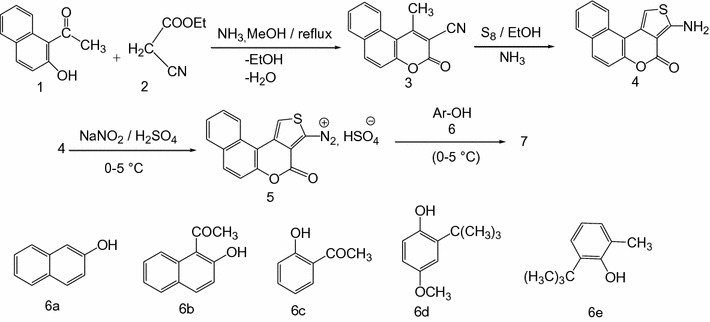
Reactions’ sequences to compounds **7**

The first step in the preparation of the coupling components was the synthesis of the relevant 2-aminothiophene **4** using the Gewald reaction [30, 31]. The synthesis of the thienocoumarins **4** from the multicomponent condensation of ketones, cyanoacetate and elemental sulphur was originally published early (Scheme 1) [25].

**Fig. 1:**
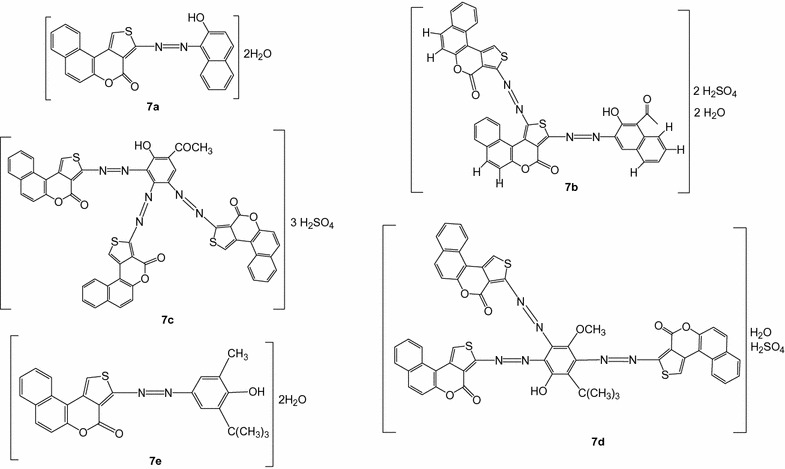
Structures of the reactions’ products **7**

Compound **4** was diazotized using nitrosyl sulphuric acid in the cold and coupled with the phenolic compounds **6a–e** to yield the azo dyes **7a–e (**Scheme 1 and Fig. 1) as previously described [24].

### Redox behaviors of the azo dyes

#### Compound 7a

**Scheme 2:**
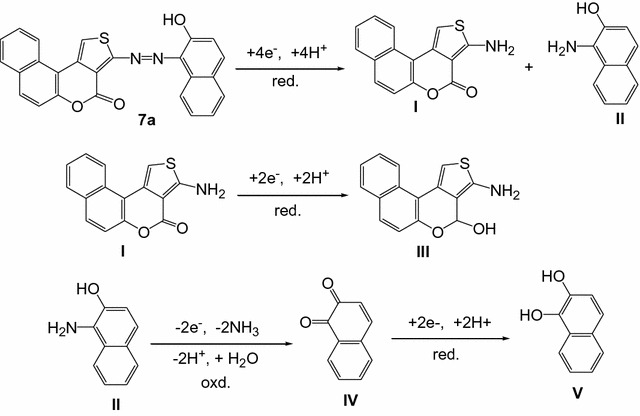
Reduction and oxidation mechanisms of compound **7a** and subsequent intermediates

Two distinct reduction peaks (Ic and IIc) were observed for the electroreduction of azo dyes **7a**, the first one Ic at 0.0046 mv due to the cleavage of the azo group, –N=N– to give the reductive amines products **I** and **II** (Scheme 2). The second peak IIc at 0.3 mv, due to the reduction of C=O group of the intermediate **I** to CH_2_OH in product **III** (Scheme 2). Since the –N=N– group is more susceptible to reduction than the C=O groups, –N=N– group is reduced at less negative potential than other sites [32].

**Fig. 2:**
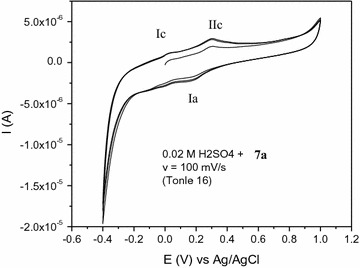
Cyclic voltammogram of 1.2 × 10^−3^M **7a** in 0.02 M sulfuric acid

The highly reactive intermediate product **II** provide quasi reversible oxidation–reduction peaks (Fig. 2) during reverse and subsequent forward scans due to the formation of oxidation product, 1,2-naphthaquinone **IV** and its subsequent reduction to dihydroxynaphthalene **V** (Scheme 2).

#### Compound 7b

**Fig. 3:**
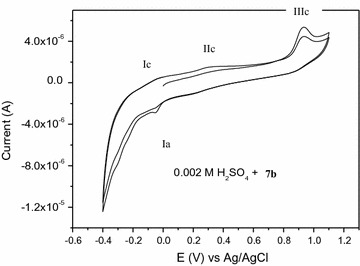
Cyclic voltammogram of 2 × 10^−3^M **7b** in 0.02 M sulfuric acid

**Scheme 3:**
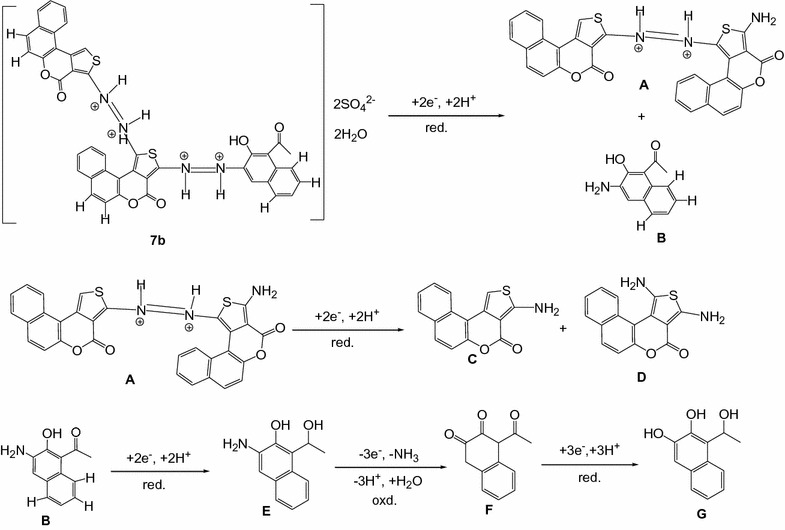
Reduction and oxidation mechanisms of compound **7b** and subsequent intermediates

In the cyclic voltammograms of **7b** (Fig. 3), four peaks were recorded, of which three cathodic peaks (Ic, IIc and IIIc) in the forward scan and one anodic peak (Ia) in the reverse scan, indicating the quasi-reversible electrochemical nature of the dye (Fig. 3). The anodic peak only appeared in the subsequent scan after the reduction step. Hence, this peak was obviously due to the corresponding oxidation of the reduction products. As reported in previous literatures [33], azo dyes with a hydroxyl group adjacent to an azo bridge can be reduced to yield the corresponding amine, which is most likely to be reoxidized in the return scan. The first peak (− 0.045 V) can be therefore attributed to the reduction of the –N=N– bridge adjacent to the hydroxyl group (Scheme 3).

The second peak (0.27 V) can therefore be attributed to the reduction of the second –N=N– bridge of compound **A**. The last peak (0.936 V) may be attributed to the catalytic hydrogen reduction of the carbonyl group (C=O) of the intermediate **B** to give compound **E** (scheme 3). The highly reactive intermediate product **E** provides a quasi reversible oxidation–reduction peaks (− 0.045 V) during reverse and subsequent forward scans (scheme 3).

#### Compound 7c

**Fig. 4:**
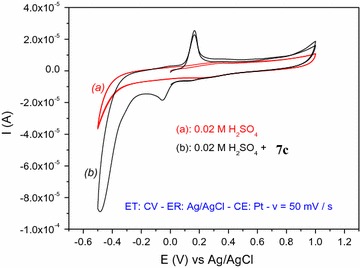
Cyclic voltammogram of 2 × 10^−3^M **7c** in 0.02 M sulfuric acid

To understand the electrochemical behavior of dye **7c**, the CV studies were carried out using solution with and without dye taking Ag wire as working electrode (Fig. 4). The potential scan used for the study was − 0.5–1.0 V. The dye solution, both showed single anodic peak approximately at − 0.0526 V and also one cathodic peak at approximately 0.168 V. The voltammetric curve of compound **7c** showed that the reduction takes place in one step and one irreversible cathodic wave was observed in cyclic voltammogram (Fig. 4).

**Scheme 4:**
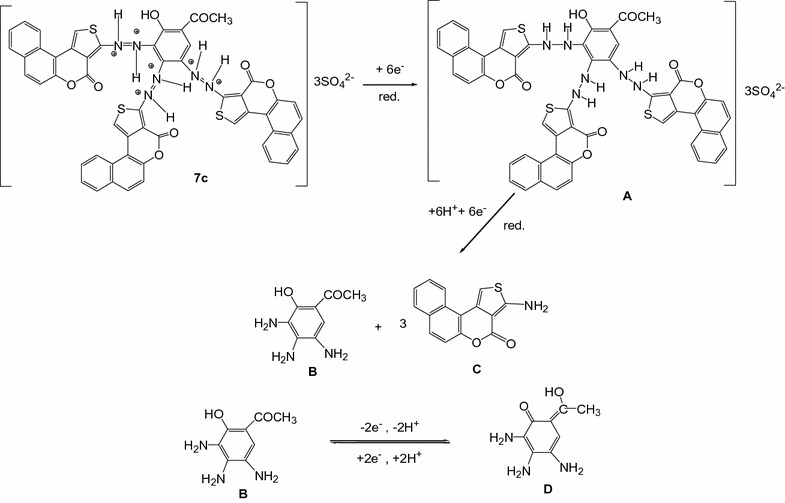
Reduction and oxidation mechanisms of compound **7c** and subsequent intermediates

The anodic peak is due to the reduction of compound **7c** to compounds **B** and **C** through the intermediate **A**. The first step of the reduction process does not however require external supply of protons, because the starting reagent **7c** is pre-protonated by the sulfuric acid crystallites. The clivage of the three azo bridges in the second step of the reduction requires six protons to yield compounds **B** and **C**. Intermediate B subsequently undergoes a quasi-reversible oxidation–reduction process during reverse and subsequent forward scans. The probable mechanism for the reduction process is displayed in scheme 4.

#### Compound 7e

**Fig. 5:**
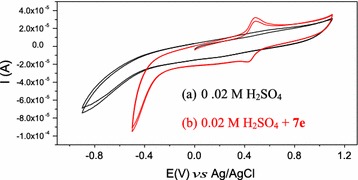
Cyclic voltammograms of 2 × 10^−3^M **7e** in 0.02 M sulfuric acid

The voltammetric behavior of compound **7e** was studied (Fig. 5). The single cathodic wave observed on the voltammograms of **7e** apparently corresponds to the reduction of the azo group and appeared in the range 0.4–0.6 mV.

**Scheme 5:**
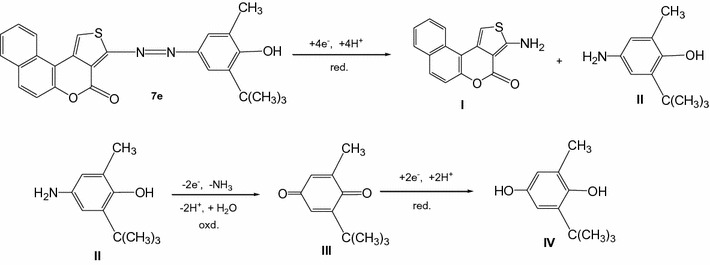
Reduction and oxidation mechanisms of compound **7e** and subsequent intermediates

The reduction process results in the clivage of the azo bridge leading to the formation of compounds **I** and **II** (Scheme 5). The intermediate **II**, further undergoes oxidation to afford compound **III** which in turn is reduced to give compound **IV** during reverse and subsequent forward scan.

### Antimicrobial activity

The azo compounds **7a–e** and the entire precursors **1–4** and **6a–e** were examined in vitro against bacterial and fungal species and the results are depicted in Table 1. All the compounds showed different degree of antimicrobial activities against the tested fungal and bacterial pathogens. *Enterobacter aerogenes* and *E. coli* were the most sensitive microorganisms while *Trichophyton terrestre* and *Trichophyton violaceum* were the most resistant. In general, bacterial species were more sensitive than fungal species; this can be due to the structural complexity of fungi compared with that of bacteria.

**Table 1:** Minimum Inhibitory Concentrations (MIC) and Minimum Microbicidal Concentrations (MMC) (µg/ml) of azo compounds and their entire precursors against fungal and bacterial strains

Microorganisms	Inhibition parameters	**3**	**4**	**6a**	**6b**	**6c**	**6d**	**6e**	**7a**	**7b**	**7c**	**7d**	**7e**	Reference drugs^a^
*Providencia stuartii* ATCC29916	MIC	> 256	> 256	128	> 256	> 256	64	> 256	16	256	32	128	32	2
	MBC	nd	nd	> 256	nd	nd	256	nd	32	> 256	64	256	32	2
	MBC/MIC	nd	nd	nd	nd	nd	8	nd	2	nd	2	2	1	1
*Escherichia coli* ATCC10536	MIC	32	128	128	32	32	64	64	4	32	32	16	16	8
	MBC	128	256	256	128	128	128	128	8	> 256	128	64	64	8
	MBC/MIC	4	2	2	4	4	2	2	2	nd	4	4	4	1
*Enterobacter aerogenes* ATCC13048	MIC	32	128	128	32	128	128	128	8	128	32	32	16	4
	MBC	128	256	256	64	128	128	> 256	8	> 256	64	128	64	4
	MBC/MIC	4	2	2	2	1	1	nd	1	nd	2	4	4	1
*Pseudomonas aeruginosa* ATCC27853	MIC	64	256	256	64	256	64	128	16	256	32	128	16	2
	MBC	128	> 256	256	128	256	64	256	32	> 256	128	256	32	2
	MBC/MIC	2	nd	1	2	1	1	2	2	nd	4	2	2	1
*Klebsiella pneumoniae* ATCC11296	MIC	128	256	128	128	> 256	64	> 256	16	> 256	32	256	16	4
	MBC	256	> 256	256	256	nd	128	nd	32	nd	128	> 256	32	4
	MBC/MIC	2	nd	2	2	nd	2	nd	2	nd	4	nd	2	1
*Staphylococcus aureus*	MIC	256	256	256	256	64	64	256	16	128	32	64	32	4
	MBC	256	> 256	> 256	256	256	64	> 256	64	> 256	128	256	64	4
	MBC/MIC	1	nd	nd	1	4	1	nd	4	nd	4	4	2	1
*Trichophyton terrestre* E1501	MIC	256	> 256	> 256	> 256	> 256	128	> 256	16	> 256	32	256	16	4
	MFC	> 256	nd	nd	nd	nd	256	nd	16	nd	64	256	64	8
	MFC/MIC	nd	nd	nd	nd	nd	2	nd	1	nd	2	1	4	2
*Trichophyton violaceum*	MIC	> 256	> 256	128	> 256	> 256	64	> 256	8	> 256	64	128	32	4
	MFC	nd	nd	256	nd	nd	256	nd	16	nd	128	256	128	8
	MFC/MIC	nd	nd	2	nd	nd	4	nd	2	nd	2	2	4	2
*Trichophyton ajeloi*	MIC	256	> 256	16	256	> 256	128	256	8	256	32	128	32	4
	MFC	256	nd	64	256	nd	256	> 256	8	> 256	64	256	128	4
	MFC/MIC	1	nd	4	1	nd	2	nd	1	nd	2	2	4	1
*Candida parapsilosis* ATCC22019	MIC	64	> 256	128	> 256	> 256	128	> 256	4	64	16	64	16	2
	MFC	128	nd	128	nd	nd	> 256	nd	8	128	32	64	32	2
	MFC/MIC	2	nd	1	nd	nd	nd	nd	2	2	2	1	2	1
*Candida albicans* ATCC9002	MIC	128	> 256	> 256	> 256	> 256	128	> 256	4	64	16	64	16	4
	MFC	128	nd	nd	nd	nd	> 256	nd	8	128	32	64	64	4
	MFC/MIC	1	nd	nd	nd	nd	nd	nd	2	2	2	1	4	1
*Cryptococcus neoformans* IP95026	MIC	32	> 256	32	> 256	> 256	128	> 256	2	256	16	32	8	4
	MFC	64	nd	64	nd	nd	256	nd	2	> 256	32	64	16	4
	MFC/MIC	2	nd	2	nd	nd	2	nd	1	nd	2	2	2	1

^a^Ciprofloxacin for bacteria, Griseofulvin for dermatophytes and Nystatin for yeasts; nd : not determined; compounds **1** and **2** were not active against all the tested microorganisms at concentrations up to 256 µg/ml

No activity was noted with compounds **1** and **2** against all the tested microorganisms (not shown). However, the Knoevenagel condensation [34] of **1** and **2** afforded the coumarin intermediate **3** which exhibited a relatively higher antimicrobial activity. Moreover, diazotisation of compound **4** with nitrosyl sulphuric acid and coupling with phenol derivatives resulted into an effective enhancement of the antimicrobial activity in compounds **7a,c–e**. Compounds **6a–e** and **7a–e** showed selective activities; their inhibitory effects being noted respectively on 10/12 (83.33%), 6/12 (50.00%), 4/12 (33.33%), 12/12 (100.00%), 5/12 (41.66%) and 12/12 (100%), 9/12 (75.00%), 12/12 (100%), 12/12 (100.00%), 12/12 (100%) of the studied microorganisms. Compounds **6d** and **7a,c–e** showed antimicrobial properties against all the tested microorganisms (MIC = 2–256 µg/ml). This finding suggests the antibacterial and antifungal potencies of these compounds. The lowest MIC value for these tested compounds (2 µg/ml) was obtained with compound **7a** on *Cryptococcus neoformans*. The antimicrobial activities of compound **7a** (MIC = 2–16 µg/ml) were in some cases equal or more important than those of ciprofloxacin (MIC = 2–8 µg/ml) and nystatin (MIC = 2–4 µg/ml) used as reference drugs; highlighting its good antimicrobial potency. The results of the MMC values indicate that most of them are not more than fourfold their corresponding MICs. This proves that the killing effects of many tested compounds could be expected on the most sensitive strains [35].

The present study highlighted the antimicrobial activity of the azo compounds and their precursors against the microorganisms including bacterial and fungal species. Although azo compounds have been reported to possess interesting activity against a wide range of microorganisms [35–37], no study has hitherto been reported on the activity of the azo dyes **7a–e** and their precursors **3**, **4** and **6a–e** against these types of pathogenic strains. As far as the structure–activity relationship is concerned, some structural features that might have influenced the antimicrobial activity of these azo compounds can be drawn from the comparison of the chemical structures of the screened compounds with different activities. Compound **7a** was the most active azo compound, followed by **7e**, **7c**, **7d** and **7b**. It appears that, in general, hydroxyl, 2-tertbutyl, 4-methoxy and aromatic groups play a greater role in increasing the antimicrobial activity based on the substitution patterns of the aromatic rings.

### Effects of azo functionality to the activity of compounds 7

It results from Table 1 that the microbicidal activity of compound **7c** on *P. stuartii ATCC29916, Klebsiella pneumoniae* ATCC11296, *Trichophyton terrestre* E1501, *Trichophyton violaceum, Trichophyton ajeloi, Candida parapsilosis, Candida albicans* ATCC 9002 and *Cryptococcus neoformans* IP95026 is entirely due to the presence of azo groups in the molecule. The microbicidal activity of compound **7e** on *Providencia stuartii ATCC29916, Klebsiella pneumoniae* ATCC11296, *Staphylococcus aureus, Trichophyton terrestre* E1501, *Trichophyton violaceum, Trichophyton ajeloi, Candida parapsilosis, Candida albicans* ATCC 9002, *Candida parapsilosis* ATCC 22019 and *Cryptococcus neoformans* IP95026 is also attributed to the presence of the azo function in the molecule. Conversely, it was noted that the azo functionality inhibited the activity on *E. coli* ATCC10536 and *Enterobacter aerogenes ATCC13048* with the transformation of the starting materials **4** and **6b** into compound **7b**. These observations corroborate previous reports related to the role played by the azo function in similar biological active substances [38].

## Conclusion

Thienylazoaryls compounds **7a–e** were synthesized, studied electrochemically at a glassy carbon electrode and preliminarily evaluated for their in vitro antimicrobial properties. The reduction of the azo group in compounds **7** exhibited different behavior due to the constitutional structure of the dyes. It was observed that pre-protonated forms get involved in the reduction step and a different number of protons are involved. The protonation reaction was facilitated owing to the increasing electron density of the azo group, due to the donating effect of the hydroxyl group at the *ortho* position. Then, a decrease in the electron density on electroactive functional group led to an easy reduction process. Compounds **7a,c–e** as well as their precursors **3** and **6d** displayed good antibacterial and antifungal activities. The presence of hydroxyl, 2-tertbutyl, 4-methoxy and aromatic groups could explain their good antibacterial and antifungal activities. Further studies are needed to determine additional physicochemical and biological parameters in order to provide a deeper insight into the structure–activity relationship and to optimize the potentials of these compounds.

## Abbreviations

T.L.C: thin layer chromatography
IR: infra-red
UV: ultra-violet
HREIMS: high resolution electron impact mass spectrometry
^1^H-NMR: proton nuclear magnetic resonance
13C-NMR: thirteen carbon nuclear magnetic resonance
DMSO: dimethylsulfoxide
TMS: tetramethylsilane
mp: melting points
THF: tetrahydrofuran
MIC: minimum inhibitory concentration
MBC: minimum bactericidal concentration
MFC: minimum fungicidal concentration
MHB: Mueller-Hinton Broth
SDB: sabouraud dextrose broth
MMC: minimum microbicidal concentration
CV: cyclic voltammogram
